# Genomic Imprinting in the New Omics Era: A Model for Systems-Level Approaches

**DOI:** 10.3389/fgene.2022.838534

**Published:** 2022-03-15

**Authors:** Jean-Noël Hubert, Julie Demars

**Affiliations:** GenPhySE, Université de Toulouse, INRAE, ENVT, F-31326, Castanet Tolosan, France

**Keywords:** imprintome, allele-specific expression, differentially methylated region, imprinted control region, noncoding RNA

## Abstract

Genomic imprinting represents a noteworthy inheritance mechanism leading to allele-specific regulations dependent of the parental origin. Imprinted loci are especially involved in essential mammalian functions related to growth, development and behavior. In this mini-review, we first offer a summary of current representations associated with genomic imprinting through key results of the three last decades. We then outline new perspectives allowed by the spread of new omics technologies tackling various interacting levels of imprinting regulations, including genomics, transcriptomics and epigenomics. We finally discuss the expected contribution of new omics data to unresolved big questions in the field.

## Introduction

**FIGURE 1:**
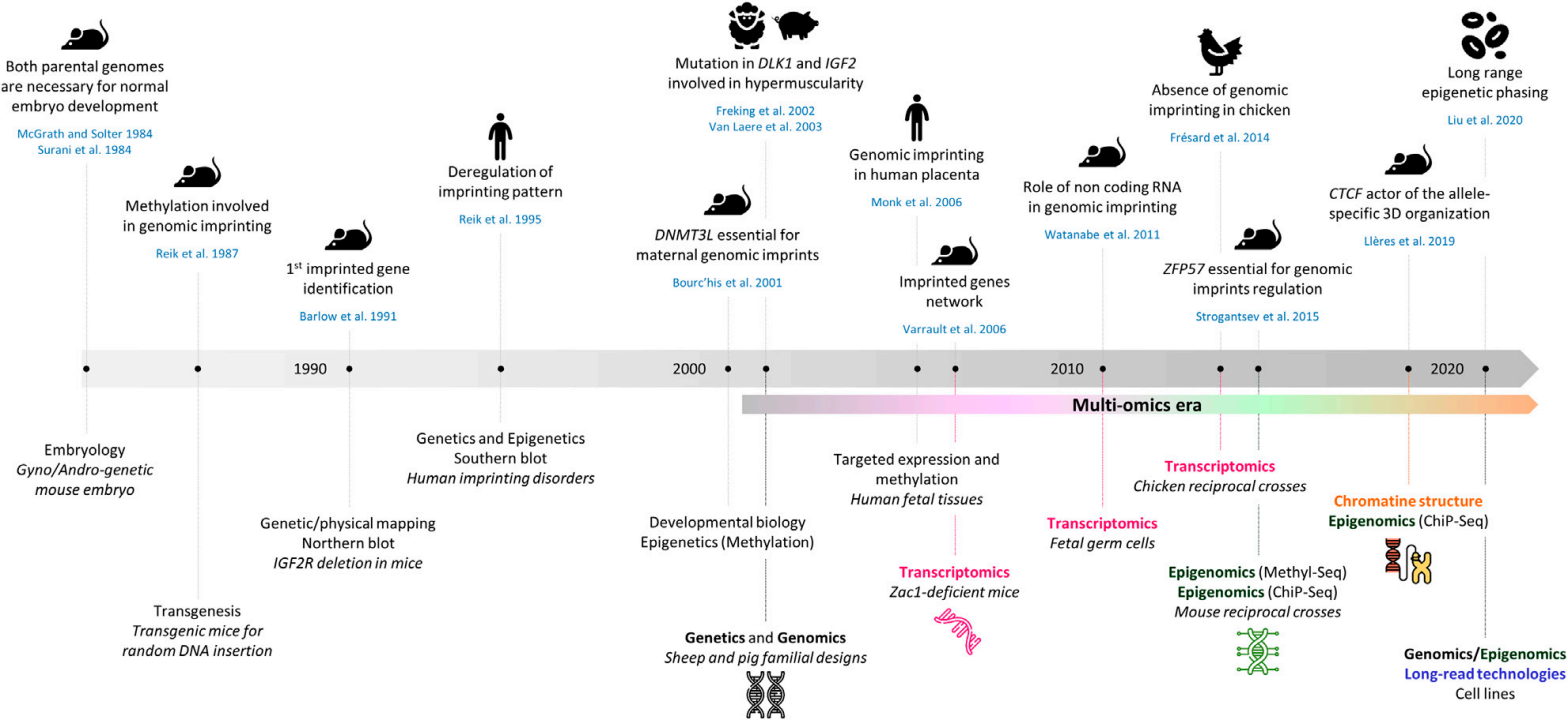
Comparative timeline of key discoveries in genomic imprinting and novel high-throughput sequencing technologies. The references cited in the figure are also present in the text unless they are mentioned hereafter (Surani et al., 1984; Watanabe et al., 2011; Strogantsev et al., 2015).

Mammals inherit two sets of chromosomes, one from each parent, and therefore possess two copies of each gene. For the majority of these genes, both alleles are expressed or repressed, depending upon the cell type. However, a little less than 1% of mammalian genes are imprinted, which means these are monoallelically expressed in a parent-of-origin (PofO)-specific manner. Since the discovery of genomic imprinting (GI) in the 80s, this field of biology was observed from different angles to better understand the originality of this mode of inheritance (Figure 1).

In brief, (i) many works have tackled the origin and the dynamics of acquisition of this process across the development of mammals from pre-implantation to post-fertilization and beyond (Monk et al., 2019), (ii) other groups have focused on both the conservation and specificity of these mechanisms across phylogeny, developmental stages and tissues (Monk, 2015; Patten et al., 2016; Edwards et al., 2019), (iii) a growing body of research has determined the key role played by imprinted genes in biological functions, physiological processes and diseases in humans (Peters, 2014; Tucci et al., 2019), (iv) thanks to the exceptional progress of knowledge in epigenetics over the last 15 years, significant advances were made on the molecular mechanisms at play through the study of GI as an example of epigenetic regulation. These achievements have benefited from the growing variety, volume and availability of omics data. Current and future applications include sequence-based analyses encompassing the many types of molecules and interactions involved in GI to get a more comprehensive and accurate view of such an epigenetic phenomenon.

In the present review, we offer a quick overview of the first three aspects mentioned above, completed by a more developed part on the molecular mechanisms involved in GI through epigenetic marks, noncoding RNAs and chromatin organization. Building on this, we introduce why and how novel sequencing technologies and multi-omics approaches will help tackle the study of GI genome-wise.

## GI is a Multistep Process and Imprints Need to Be Reset at Each Generation

The identification of the first imprinted genes (Barlow et al., 1991; Rachmilewitz et al., 1992) sparked initial efforts towards elucidating the mechanisms of imprint establishment, maintenance and erasure (Morgan et al., 2005; Ferguson-Smith and Bourchis, 2018; Monk et al., 2019). In primordial germ cells (PGC), the genome undergoes extensive DNA demethylation, including the removal of existing previous parent-specific imprints. New imprints are acquired at later stages of gametogenesis, according to the sex of the embryo, with a sex-specific timeline. In sperm, imprint establishment starts before birth and is completed in perinatal period, whereas in the female germline imprints are acquired after birth, during oocyte growth (Lucifero et al., 2004; Kato et al., 2007). Those germline imprints, known as primary imprints, are left on specific regions called Imprinting Control Regions (ICRs), which are the site of key imprinting regulations. *DNMT3A* and its cofactor *DNMT3L* are the main genes involved in the *de novo* methylation activity in both germlines (Bourc’his et al., 2001; Kato et al., 2007). *KDM1B*, which encodes a lysine demethylase almost exclusively expressed in growing oocytes, is critical for establishing several maternal imprints during oogenesis, as well as non-histone transcriptional regulators, including *ZFP57* and *NLRP2* among others (Begemann et al., 2018; Ferguson-Smith and Bourchis, 2018). *ZFP57* has further post-fertilization role, when imprinted regions of paternal and maternal germline withstand a wave of genome-wide demethylation followed by a wave of *de novo* methylation. In addition, *DNMT1* is crucial to maintain the methylation imprints in the preimplantation embryo (Hirasawa et al., 2008). Other key regulators of the maintenance of GI include in particular *DPPA3* (Nakamura et al., 2007), *CTCF* (Engel et al., 2006) and components of the nucleosome remodeling and histone deacetylation (NuRD) complex, such as *MBD3* (Reese et al., 2007) and *MTA2* (Ma et al., 2010).

## Conservation of Imprinting Patterns Across Mammals and Between Tissues Occurs Restricted

Approximately 200 imprinted genes have been documented to date in humans and mice. In other species, a few dozen loci have been experimentally validated at most, such as in rats and pigs with 14 and 45 imprinted genes identified to date, respectively (http://www.geneimprint.com, last accessed January 2022). Several studies suggested that imprinted genes were less conserved across mammals than initially thought (Monk et al., 2006; Khatib et al., 2007). Genes of the Kcnq1 cluster found to be imprinted in the mouse placenta are not in humans (Monk et al., 2006), while the opposite was shown for *L3MBTL* (Li et al., 2005). In a more complex way, *IGF2R* was imprinted in the mouse but exhibited a polymorphic, variable imprinting pattern in humans (Xu et al., 1993). These findings suggest that GI differs between mammals and displays species-specific regulation patterns, raising questions on the conservation of ICRs across species. Interestingly, the placenta is the tissue with the most imprinting discrepancies between the mouse and humans (Monk, 2015). Genome-wide analyses showed most of the imprinted clusters with differentially methylated regions (DMRs) in the human placenta are not differentially methylated in the mouse placenta (Miri et al., 2013; Court et al., 2014), which suggests that widespread differences have occurred during imprinting evolution. Additionally, many imprinted genes exhibit brain-specific functions and expression patterns. A textbook case is *UBE3A*, which shows a biallelic expression in most tissues but a maternal expression profile within certain neuronal subtypes (Albrecht et al., 1997). In a more complex way, *IGF2* is paternally expressed in the subgranular zone of the hippocampus, acting as an autocrine factor, but biallelically expressed in the subventricular area, displaying a paracrine role (Ferrón et al., 2015). Such functionally important mechanisms of transcriptional dosage control highlight the shape-shifting nature of GI across cells and tissues.

## Imprinted Genes are Key Regulators of Fetal and Post-Natal Growth and Adult Behaviour

The discovery of the crucial roles of imprinted genes came from uniparental mouse embryos and then from human imprinting disorders (Peters, 2014). Experimentally-produced uniparental embryos show lethality due to aberrant GI patterns in several species including cattle, sheep and pig (Lagutina et al., 2004; Zacchini et al., 2011; Sembon et al., 2012), which is in line with pioneering studies showing development arrest due to a lack of embryonic or extraembryonic tissues in the mouse (Barton et al., 1984; McGrath and Solter, 1984). Paternally-expressed *IGF2* is a well-studied example of imprinted gene that positively regulates fetal growth (DeChiara et al., 1991; Ferguson-Smith et al., 1991). Oppositely, maternally-expressed *GRB10* acts as an essential growth restrictor (Shiura et al., 2009). It has been proposed that many imprinted genes contributing to growth control pathways are coordinately regulated in multiple tissues within an imprinted gene network (Varrault et al., 2006). As suggested through the contribution of GI to growth-related phenotypes, imprinting dysregulation has been identified in a set of 13 so-called imprinting disorders harbouring convergent patterns of molecular alterations and clinical features (Eggermann et al., 2021). Imprinted genes also have a long-known and important role in the development of the mammalian brain and in adult behaviour, which is illustrated by the contributions of *PEG1* and *PEG3* to maternal behaviour (Ho-Shing and Dulac, 2019; Tucci et al., 2019).

## GI is a Particularly Attractive Example of Epigenetic Regulation

### Main Mechanistic Features of GI

Epigenetics relates to stable and heritable patterns of gene expression that do not involve changes in DNA sequence. GI is a particularly attractive example of epigenetic regulation leading to PofO-specific gene expression (Bartolomei et al., 2020), since in the same cell only one of the two parental alleles is stably repressed depending on epigenetic marks (Reik and Lewis, 2005). Imprinted genes are typically located in clusters of 3–12 genes that are spread over 20 kb–3.7 Mb of DNA, although examples of single imprinted genes do exist (Edwards and Ferguson-Smith, 2007). Clusters of imprinted genes, designated as imprinted domains, harbor biallelically-expressed genes alongside maternally- and paternally-expressed genes, which encode both protein-coding and long noncoding (lnc) RNAs. Each cluster carries an ICR exhibiting PofO-specific epigenetic marks, such as DNA methylation and post-translational histone modifications, which differentially tag the parental alleles as either active or repressed (Maupetit-Méhouas et al., 2016). A textbook example is the well-known Igf2/H19 imprinted cluster (Nativio et al., 2011). PofO-specific DNA methylation occurring at ICRs, also called canonical imprinting, is considered a primary imprint marker that directly or indirectly controls most of imprinted genes (Kobayashi, 2021). These primary imprints are germline differentially methylated regions (gDMRs) that are maintained after fertilization. In addition, some PofO-specific DNA methylations are set post-zygotically in somatic lineages (sDMRs) and are considered as secondary imprints (Kobayashi, 2021). Memory mechanisms allowing the PofO-specific DNA methylation after the global erasure are yet to be discovered. Noncanonical imprinting has been identified as another key gametic imprinting mark mediated by maternal histone modification instead of DNA methylation (Chen et al., 2021; Mei et al., 2021).

### Long-Range Regulations in Imprinted Clusters

Two well-defined mechanisms of imprinted gene regulation have been described so far: the insulator model and the lncRNA model (Plasschaert and Bartolomei, 2014). The insulator model is best illustrated at the Igf2/H19 locus. In this example, ICRs work as chromatin insulators and control the reciprocal imprinting of both maternally-expressed *H19* and paternally-expressed *IGF2* through the differential allelic binding of the CTCF protein. In fact, CTCF binds to the unmethylated maternal ICR and forms an insulator, preventing *IGF2* expression for the benefit of the H19 lncRNA. On the paternal allele, the hypermethylated ICR prevents CTCF from binding and the insulator from forming, which allows the downstream enhancers to promote *IGF2* instead of *H19*. The lncRNA model is depicted by the Igf2r/Airn locus, in which the promoter of a lncRNA is located within the ICR. This allows the activation of the lncRNA from the unmethylated paternal ICR, silencing the adjacent genes in *cis*. Silencing is mediated through either the attraction of the machinery that lay down repressive chromatin marks (Nagano and Fraser, 2009) or the prevention of the RNA polymerase II recruitment at promoters (Latos et al., 2012). On the maternal allele, the hypermethylated ICR results in silencing the lncRNA, thereby allowing the activation of neighboring genes.

### GI as Part of Coregulated Networks

Systems-level approaches to GI have increasingly developed since the demonstration showing that the perturbation of one imprinted gene may affect other imprinted genes as well as biallelically-expressed genes (Varrault et al., 2006; Gabory et al., 2009). Therefore, an imprinting gene network (IGN) involving several imprinted genes and non-imprinted genes was suggested (Patten et al., 2016). First studies have confirmed that many imprinted genes are indeed coregulated in their expression levels (Varrault et al., 2006; Al Adhami et al., 2015). Interestingly, in porcine fetal liver cells, a sub-network involving *IGF2*, *DLK1* and *MEG3* was shown using 3D Fluorescence *in situ* Hybridization (FISH), suggesting that 3D nuclear organization, through the colocalization of these imprinted genes, is linked to their transcriptional state (Lahbib-Mansais et al., 2016). While the *cis*-regulation of different imprinted genes, often through the repressive role of imprinted lncRNAs, is well documented, more and more studies have revealed *trans*- silencing mechanisms (Ghousein and Feil, 2020; Whipple et al., 2020). In the *Dlk1-Meg3* imprinted region, a dense cluster of 39 miRNAs, miR-379/410, is located in the 3′UTR of maternally-expressed *MEG3*. Such maternal miRNAs downregulate several paternally-expressed genes located elsewhere like *PLAGL1* (Whipple et al., 2020), which directly regulates itself a few hundred covarying genes, including multiple imprinted genes, together constituting a gene network (Varrault et al., 2017).

## Towards Characterizing Imprintomes in the New Omics Era

**FIGURE 2:**
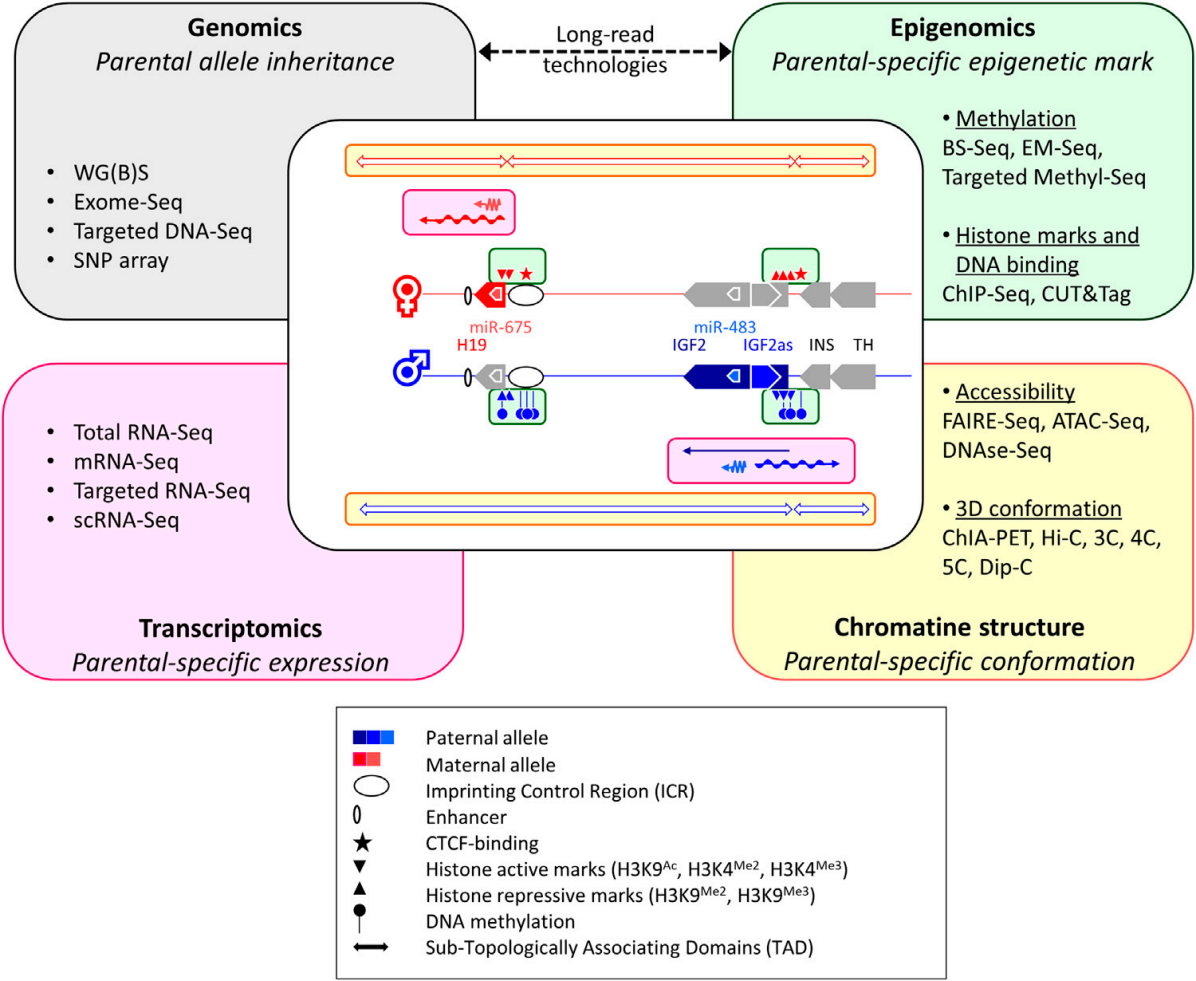
How multi-omics nurse knowledge on genome imprinting mechanisms. The diagram in the center of the figure depicts the different layers of GI regulation that can be targeted using current multi-omics, through the canonical example of the Igf2/H19 imprinted cluster. The colored boxes used in the central diagram (green, pink and yellow) represent the different interacting levels of imprinting regulations that are mentioned in the corresponding omics boxes. WG(B)S: Whole-Genome Bisulfite Sequencing; BS-Seq: BiSulfite-Sequencing; EM-Seq: Enzymatic Methyl-Sequencing; ChIP-Seq: Chromatin ImmunoPrecipitation followed by Sequencing; CUT&Tag: Cleavage Under Targets and Tagmentation; scRNA-Seq: single-cell RNA-Seq; FAIRE-Seq: Formaldehyde-Assisted Isolation of Regulatory Elements Followed by Sequencing; ATAC-Seq: Assay for Transposase-Accessible Chromatin with high-throughput Sequencing; ChIA-PET: Chromatin Interaction Analysis with Paired-End Tag sequencing, 3C: Chromosome Conformation Capture; 4C: 3C on Chip; 5C: 3C-Carbon Copy; HiC: High-throughput 3C; Dip-C: Diploid 3C.

Beside an accurate understanding of the molecular regulation of the different imprinting regions, acquiring a global overview of the imprinted gene network remains crucial to better apprehend their major roles genome-wise. In this context, recent developments in omics (including genomics, transcriptomics, epigenomics and chromatin structure analyses, see Figure 2) should provide more and more comprehensive insights on the role of GI in complex traits in mammals (O’Doherty et al., 2015) and human disorders (Monk et al., 2019).

### *Via* (Epi)Genomics

Investigating GI requires considering an extra state of DNA sequence through the mapping of methylated cytosines. It has therefore fueled the continued development of sequencing protocols including the bisulfite conversion of unmethylated cytosines (Clark et al., 2006; Olova et al., 2018), so that both conventional genotype information and the methylation status of cytosines in any sequence context can be jointly determined. As the cost of acquiring full sequence data has decreased, Whole-Genome Bisulfite Sequencing (WGBS) has emerged as a standard to move towards more exhaustive maps of GI in species with a reference sequence assembly (Zhou et al., 2021). Today there is a rich set of library preparation strategies using short-read technologies to implement genome scans for imprinted genes, from affordable ones based on methylation-dependent restriction enzymes and suited for *de novo* analyses (Wang et al., 2015; Dixon and Matz, 2021) to bisulfite-free ones aimed at preserving DNA sequence integrity while seeking exhaustiveness (Liu et al., 2020; Vaisvila et al., 2021). Genome-wide analyses of parent-offspring trios, reciprocal crosses and other pedigree-based designs have pivotal importance in detecting molecular signatures of GI (Frésard et al., 2014; Zink et al., 2018; Consortium et al., 2019). Such studies have much to gain from the use of long-read technologies from Oxford Nanopore and PacBio platforms, which are able to read both DNA sequence and its methylation status over several kilobases. Long-range phasing will in particular improve the acquisition of the PofO information. More generally, long reads should improve several facets of GI studies, including allele-specific variant detection, access to complex sequence and parental methylation bias identification (Gigante et al., 2019). This paves the way to generalizable approaches coupling affordable pedigree-based designs with low-coverage long-read sequencing data, which could become essential to improving our understanding of GI. Interestingly, combining bisulfite-free sequencing library preparation strategies with long reads is appealing both in theory and in practice (Liu et al., 2020), making it possible to envisage many beneficial applications for the better characterization of GI. By allowing the genome-wide detection of protein-DNA interactions and histone modifications, Chromatin ImmunoPrecipitation followed by Sequencing (ChIP-Seq) offers additional possibilities to investigate the mechanistic features of GI, including noncanonical patterns (Chen et al., 2021; Mei et al., 2021).

### *Via* Transcriptomics

As imprinting mechanisms are organized at the scale of transcriptional units, we anticipate that current developments in transcriptomics bring much to our understanding of GI, in particular through the spread of single-cell RNA-Seq (scRNA-Seq) experiments and long-read technologies. scRNA-Seq allows measuring gene expression at the cell resolution, which is particularly relevant to characterize imprinted genes with tissue- or cell-specific expression patterns. Imprinting expression patterns may vary from mono- to biallelic across cells, suggesting the occurrence of epigenetic mosaicism in mammals (Ginart et al., 2016). scRNA-Seq experiments are here both highly advisable and challenging because the tissues most subjected to GI show remarkable spatial and temporal heterogeneity still undergoing exploration (Liu et al., 2018; Varrault et al., 2020). First studies showed the potential of scRNA-Seq to identify new imprinted candidates (Santoni et al., 2017) and to dissect the complexity of dosage imbalance phenomena in the cell (Stamoulis et al., 2019). The regulation of gene expression in imprinting clusters is provided in particular by lncRNAs, which are located in the immediate vicinity of ICRs and have an effect on large physical distances within clusters. Their precise roles need further clarification, but it is accepted that lncRNAs do more than simple transcriptional interference and are required for imprinting maintenance (MacDonald and Mann, 2020; Llères et al., 2021). As such RNAs may exceed one kilobase in length, the use of direct RNA-seq methods compatible with long reads appears an appropriate strategy to favor their characterization while limiting the occurrence of bias (Garalde et al., 2018). Current effort is focused on developing suitable methods to allow transcriptome-wide representations of long transcripts, including those without polyadenylated tails (Oikonomopoulos et al., 2020; Begik et al., 2021). Given the importance of noncoding RNA species in mediating GI, the development of new comprehensive transcriptomic approaches based on total RNA-Seq (Verboom et al., 2019), aiming at simultaneously detecting diverse RNA types, is good news for future GI studies.

### *Via* 3D Genomics

Both the epigenetic landscape and the RNA-protein complexes regulating imprinted genes are part of a bigger picture involving higher-order organization constraints in the nucleus. The development of Chromosome Conformation Capture (3C)-based technologies (C-technologies) makes it possible to study the links between nuclear architecture, chromatin topology and genetic elements, leading to genome-wide 3D maps (Rao et al., 2014). The key principle of C-technologies is to obtain the sequence information of frequently interacting chromosome fragments to identify gene regulations at the scale of the 3D nucleus (Barutcu et al., 2016). Such data confirmed the master role of CTCF in 3D genome organization, supporting the view that further characterization of the interactions between chromatin structures and molecular binding complexes in imprinted domains will shed light on mechanisms underlying the maintenance and dynamics of GI (Llères et al., 2019; Noordermeer and Feil, 2020). By jointly improving resolution, phasing and genome coverage, C-technologies have revealed specific higher-order structural patterns about GI. In particular, High-throughput 3C (Hi-C) showed the enrichment for imprinted genes in chromatin loops (Greenwald et al., 2019). Current efforts lay the foundation for identifying differences in 3D structure between maternal and paternal alleles in imprinted clusters (Tan et al., 2018; Lindsly et al., 2021). In addition, chromatin accessibility analyses like Assay for Transposase-Accessible Chromatin with high-throughput Sequencing (ATAC-Seq) or Chromatin Overall Omic-scale Landscape Sequencing (COOL-Seq) make it possible to test the existence of such a parental asymmetry at a lower level of chromatin organization (Wu et al., 2016; Gu et al., 2019). There is therefore today a dense set of high-throughput technologies for analyzing chromatin organization, from the gene-level resolution to long-range contacts, which allow genome-wide integrative analyses on the chromatin mechanisms regulating imprinted networks.

## Discussion

Review of sequence-based technological developments shows a transition taking place along two transversal axes, from bulk to single-cell approaches and from short to long reads. Such an evolution carries many promises in the context of GI studies, especially as imprinted sites host very diverse elements and are subjected to various regulatory features. It is therefore the right time to characterize in depth imprintomes and understated regulations across loci, stages, cell types and species, which will lead to a better mechanistic understanding of GI. The incorporation of C-technologies as part of multi-omics integrative approaches could in particular reveal imprinted interactomes (Naveh et al., 2021).

Analyses of coding sequences remain an essential gateway to increase our understanding of GI, as the identification of new imprinted genes leads to various further studies. Affordable genome-wide data acquisition benefiting from pedigree-based designs can be implemented across phylogenetic clades, thereby helping to address large sets of questions related to imprinting evolution. These include a better understanding of the early evolutionary history and diversification of GI. A closer look at GI in certain taxa and tissues through integrative omics approaches could for example help clarify the constraints applied to imprinted clusters, the mechanisms that enabled the acquisition of DMRs and the role of transposable elements in the evolution of mammalian development (Bogutz et al., 2019; Hanin and Ferguson-Smith, 2020; Hanna and Kelsey, 2021; Senft and Macfarlan, 2021). Comprehensive genome scans for imprinted genes in species with little or no previous evidence for GI bring important information, since it promotes the understanding of both GI evolution and related phenomena such as methylation reprogramming and allele-specific expression, which regulate key biological processes in vertebrates (Frésard et al., 2014; Zhuo et al., 2017; Skvortsova et al., 2019). A recent epigenome comparison across five placental mammals notably showed striking species-specific features, with distinct GI mechanisms between humans, nonrodents and rodents (Lu et al., 2021).

All this highlights the great interest of studying how GI may influence phenotypes across mammals. Some strategies are emerging to identify the impact of very subtle changes related to GI on intermediate molecular phenotypes (Greenwald et al., 2019; Lindsly et al., 2021). At a higher phenotypic level, we know from familial and association studies that GI contributes to complex phenotypes, including syndromic disorders (Eggermann et al., 2021), cancer (Goovaerts et al., 2018) and several other developmental phenotypes in both humans (Workalemahu et al., 2020) and other mammals (Freking et al., 2002; Van Laere et al., 2003). Investigating multi-scale GI-phenotype relationships could provide insights on unusual patterns of missing heritability, with the potential for many applications. Genomic prediction in domestic animals could for example benefit from explicitly modeling GI for some economically important phenotypes (O’Doherty et al., 2015; Hu et al., 2016; O’Brien and Wolf, 2019). In cancer, evasion of growth suppression is mediated through many imprinted loci (Stampone et al., 2018; Lecerf et al., 2019; Sutton et al., 2019). Studies on experimental models or patient tissues would be helpful to further document the contribution of dysregulated imprinting patterns to cancer evolution (Lozano-Ureña et al., 2021; Taguchi et al., 2021). More generally, imprinted clusters host key genes offering a gateway to larger epigenomic studies. We therefore believe that current developments in sequencing technologies are essential to significant advances in the characterization of such unusual modes of trait transmission.
